# Minimising the duration of N95 respirator use during hospital SARS-CoV-2 outbreaks: A mixed-effects analysis of post-screening infection reduction

**DOI:** 10.1016/j.infpip.2025.100484

**Published:** 2025-09-08

**Authors:** Mari Yanaka, Toshibumi Taniguchi, Misuzu Yahaba, Shota Murata, Hiroshi Yoshikawa, Hitoshi Chiba, Misao Urushihara, Hidetoshi Igari

**Affiliations:** a Department of Infectious Diseases, Chiba University Hospital, Japan; b Division of Laboratory Medicine, Chiba University Hospital, Japan

**Keywords:** N95 respirators, SARS-CoV-2, Nosocomial infection, Health personnel, Infection control, Disease outbreaks

## Abstract

Hospital SARS-CoV-2 outbreaks require effective interventions. We evaluated targeted universal N95 respirator use among staff from outbreak identification until screening results. Analysing 18 cluster outbreaks using generalised linear mixed models, we found that N95 respirator use was associated with a 72% reduction in new infections (*P*<0.001) compared to surgical masks. The mean duration of N95 respirator use was 5.25 days. Policy effectiveness was independent of outbreak size. Bootstrap analysis confirmed significant reduction (mean difference -3.02 cases, 95% CI: -5.98 to -0.08). Infection source showed substantial variability while ward-level variation was minimal. Short-term targeted N95 respirator use effectively controls hospital outbreaks while optimising resources and staff comfort.

## Introduction

Hospital SARS-CoV-2 outbreaks present significant risks to patients and healthcare providers, often necessitating admission restrictions and creating substantial financial and logistical challenges [1]. Although established preventive measures including vaccination, enhanced ventilation, and personal protective equipment with surgical masks [2] are commonly used, outbreaks continue to occur. N95 respirators offer superior protection compared to surgical masks but are costly and resource-intensive. Therefore, determining the scope of users and minimum necessary duration for effective use during outbreaks is crucial. This study investigates targeted universal N95 respirator use among hospital staff aimed at minimising the duration of use while effectively reducing new SARS-CoV-2 infections following ward screening.

## Methods

### Study design and setting

This prospective observational study with a before-and-after comparison was conducted at Chiba University Hospital, an 850-bed tertiary care academic medical centre in Chiba, Japan. Data were collected from January 2021 to January 2024, encompassing 18 cluster outbreaks across various hospital wards. During the study period, standard infection control measures remained consistent, including universal surgical masking, hand hygiene protocols, and patient isolation procedures. The first cluster occurred in January 2021, prior to vaccine availability. Healthcare workers received their first and second vaccine doses beginning in March 2021, with third doses administered from December 2021. For clusters occurring after December 2021, approximately 90% of hospital employees had received at least three vaccine doses.

### N95 respirator protocol

Universal N95 respirator use was defined as mandatory use of Japan Ministry of Health, Labour and Welfare-approved N95 respirators (Sakai-type Hi-Luck 350, Koken Ltd, Japan) by all healthcare personnel in affected wards, regardless of direct patient contact. All staff completed conventional fit testing training as part of routine hospital protocols. During outbreak periods, proper donning and doffing procedures were reinforced through brief training sessions.

When an outbreak was identified, defined as situations involving patients with unknown infection routes and positive cases from multiple rooms, all staff members working in the affected ward were required to wear N95 respirators continuously. Screening tests were then performed for both staff and patients. Upon receiving screening results, N95 respirator use was discontinued, with specific measures applied only to confirmed positive cases and identified close contacts.

### Statistical analysis

New SARS-CoV-2 cases detected after the screening period served as the primary outcome. We employed a generalised linear mixed model with Poisson distribution, incorporating cluster size and N95 respirator policy as fixed effects, with random effects for hospital ward and infection source to address intra-group variability. A predictive framework combined policy status, average infected individuals, infection sources, and hospital wards to analyse the policy's impact. Bootstrap resampling with 10,000 iterations assessed model robustness and quantified uncertainty through confidence intervals.

## Results

Our analysis of 18 cluster outbreaks (Figure S1) revealed significant differences between the N95 respirator and surgical mask groups (Table 1). Targeted universal N95 respirator usage was associated with approximately 72% reduction in new infections (coefficient = −1.27975, standard error = 0.38876, *P* < 0.001). The mean duration of N95 respirator use was 5.25 days (SD = 2.4, range: 1–8 days). Post-screening infections averaged 1.6 per cluster in the N95 respirator group versus 4.0 in the surgical mask group. Initial cluster size did not significantly influence new infection rates (coefficient = 0.01248, standard error = 0.05406, *P* = 0.817), suggesting policy effectiveness was independent of outbreak scale.

**Table 1:** Characteristics and outcomes of SARS-CoV-2 hospital outbreaks by respiratory protection type (January 2021–January 2024)

Characteristic	N95 respirator group	Surgical mask group	*P*-value
**Outbreak Characteristics**			
Number of clusters	8	10	-
Study period	Aug 2022–Jan 2024	Jan 2021–Nov 2023	-
Hospital wards affected^a^	Medical/Surgical/Mixed	Medical/Surgical/Mixed	-
**Cluster Size**			
Total cases per cluster, mean (SD)	17.5 (6.3)	14.6 (5.8)	0.314
Patient cases, n (%)	74 (52.9%)	82 (56.2%)	0.577
Healthcare worker cases, n (%)	65 (46.4%)	64 (43.8%)	0.661
- Physicians	8	9	-
- Nurses	45	38	-
- Other staff	12	17	-
**Infection Source**			
Healthcare workers	3	3	0.756
Visitors	2	2	
Unknown	1	3	
Multiple sources	2	2	
**Primary Outcome**			
Post-screening infections per cluster, mean (SD)	1.6 (1.4)	4.0 (2.8)	<0.001
- Staff	0.8 (0.9)	2.8 (2.1)	0.012
- Patients	0.9 (1.0)	1.2 (1.3)	0.563
**Intervention Details**			
Duration of mask use, days, mean (SD)	5.25 (2.4)	Continuous	-
Range	1–8 days	-	-
**Operational Impact**			
Admission restriction period, days, mean (SD)^b^	6.2 (2.2)	7.3 (4.4)	0.584
Range	3–10 days	3–18 days	-
**Model Results**			
Adjusted rate ratio (95% CI)	0.28 (0.14–0.57)	Reference	<0.001
Reduction in infections	0.72	-	-

SD = Standard deviation; CI = Confidence interval.

Note: *P*-values calculated using Mann-Whitney U test for continuous variables and Fisher's exact test for categorical variables. Model results from generalised linear mixed model with Poisson distribution adjusting for cluster size with random effects for ward and infection source.

a Ward categorization was complex due to mixed specialty units.

b Data available for 6/8 N95 respirator clusters and 8/10 surgical mask clusters.

Admission restriction periods showed a trend toward shorter duration in the N95 respirator group (mean 6.2 vs 7.3 days), with notably less variability compared to the surgical mask group (SD 2.2 vs 4.4 days; range 3–10 vs 3–18 days), though this difference was not statistically significant (*P* = 0.584).

**Figure 1:**
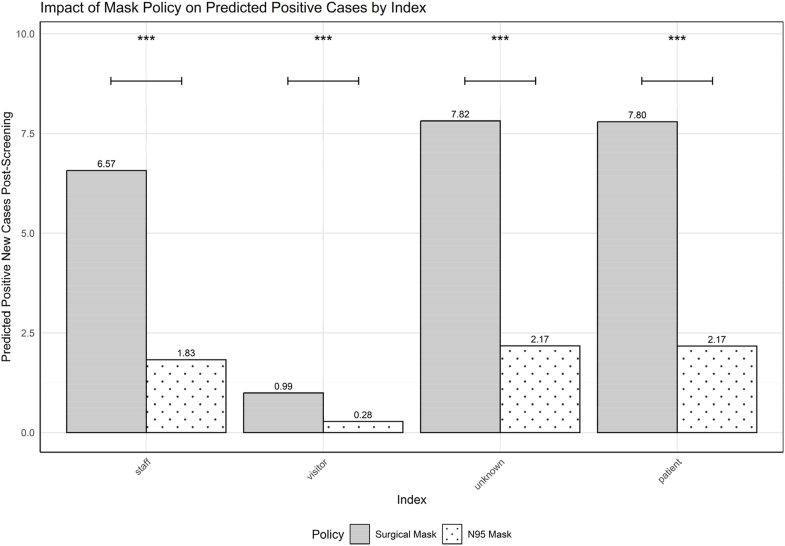
**Predicted SARS-CoV-2 cases by infection source and mask policy.** Grouped bar chart showing model-predicted positive cases following screening under surgical mask (grey bars) and universal N95 respirator (white bars with dots) policies. Data represent mean predicted cases averaged across all wards for each infection source category. Statistical significance (∗∗∗*P* < 0.001) indicates significant differences between mask policies within each infection source category based on the generalised linear mixed model analysis.

Random effects analysis indicated minimal variability in transmission dynamics across hospital wards (variance = 0.0009528), suggesting consistent transmission rates among wards. Conversely, infection initiation source exhibited significantly greater variability (variance = 0.8753182), highlighting substantial differences in transmission dynamics depending on infection source (Figure 1).

**Figure 2:**
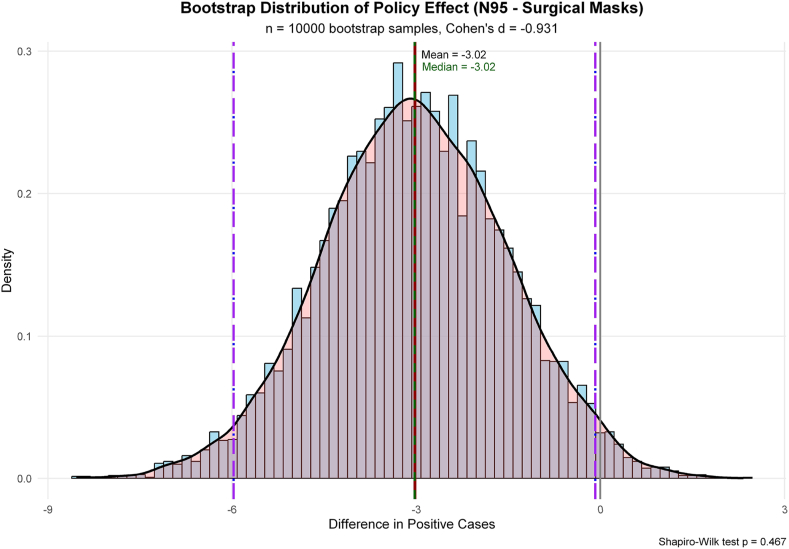
**Bootstrap Distribution of Difference in Predicted Positive Cases.** Distribution of differences in predicted SARS-CoV-2 cases between N95 respirator and surgical mask use from 10,000 bootstrap iterations. Negative values indicate fewer cases with N95 respirator use. Red dashed line: mean difference (-3.02 cases); blue dashed lines: 95% confidence interval (-5.98 to -0.08). The entirely negative distribution confirms significant reduction in infections with the N95 respirator policy.

Bootstrap analysis demonstrated significant association between N95 respirator implementation and reduction in predicted positive cases. The mean difference was −3.02 (95% CI: −5.98 to −0.08, *P* < 0.05). The probability that N95 respirator use reduces infections was 97.8%, with 74.7% probability of achieving a clinically meaningful reduction of ≥2 cases. The estimated number needed to treat was 4.3 clusters (Table S1, Figure 2).

## Discussion

Our findings demonstrate that targeted universal N95 respirator use is associated with a significant reduction in hospital SARS-CoV-2 transmission, with a 72% reduction in new infections highlighting the critical role of high-quality respiratory protection during outbreaks. Previous studies indicate healthcare workers are significant contributors to hospital-acquired infections [1,3], and N95 respirators provide bidirectional protection against transmission [4].

The policy's effectiveness was independent of outbreak size, emphasising swift implementation regardless of initial magnitude. This aligns with public health recommendations for rapid escalation of protective measures during outbreaks [5].

The minimal variation across hospital wards indicates environmental and operational factors within wards do not significantly alter N95 respirator effectiveness, consistent with studies demonstrating reliable performance of properly fitted N95 respirators across healthcare settings. The importance of proper fit testing and training, as emphasised by Lam *et al.* [6], likely contributed to consistent effectiveness. Conversely, significant variability linked to different infection sources emphasises the complex interplay between human behaviour, medical procedures, and virus transmission. This supports a nuanced approach to infection control that complements universal mask policies while addressing source-specific transmission dynamics.

A key strength is demonstrating that targeted N95 respirator use limited to 5.25 days effectively controls outbreaks while minimising adverse effects. Previous studies document skin lesions, pressure injuries, and dermatitis associated with prolonged N95 respirator use [7]. Our mean usage duration may minimise these risks compared to extended wear periods. However, we did not systematically assess skin complications or user discomfort, representing a study limitation. Future implementations should incorporate monitoring for adverse effects and consider rotating different respirator models to reduce pressure points [8].

By limiting mandatory N95 respirator use strictly to the period between outbreak identification and screening results, our approach minimises discomfort and fatigue while conserving critical PPE resources. Additionally, the trend toward shorter admission restriction periods in the N95 respirator group (6.2 vs 7.3 days) with less variability suggests potential operational benefits, avoiding prolonged restrictions seen in some surgical mask clusters. This targeted strategy enhances compliance among staff while addressing practical logistical and economic challenges associated with prolonged respirator usage.

Our findings support flexible mask policies providing high-level respiratory protection precisely when needed, optimising both healthcare worker safety and resource allocation. The conditional implementation of N95 respirator policies during outbreaks, with return to surgical masks during low-risk periods, ensures protection during critical times while addressing resource sustainability and wearer comfort [9]. Hospitals should integrate flexible mask policies into infection control strategies, swiftly transitioning between surgical masks and N95 respirators based on real-time outbreak assessment.

Future research should investigate the combined effects of N95 respirators with other preventive measures to understand collective impact on transmission [10], as well as logistical and economic aspects of implementing flexible mask policies to determine feasibility and sustainability within healthcare settings.

## Limitations

This single-centre study may limit generalisability to other healthcare environments. The observational, quasi-experimental design limits causal inference, and the temporal difference between surgical mask (predominantly early period) and N95 respirator groups (predominantly later period) may introduce time-period bias, though standard infection control measures remained consistent and, except for one early cluster in January 2021, all outbreaks occurred after March 2022 when vaccination coverage had stabilized at approximately 90% with three or more doses. We did not systematically assess adverse effects such as skin lesions, breathing difficulty, or communication challenges during N95 respirator use. The absence of healthcare worker compliance evaluation and cost-effectiveness analysis comparing resource utilisation between strategies represent additional limitations. The study could not account for potential confounding factors such as changes in other infection control measures during the study period. Limited generalisability exists for resource-limited healthcare settings where fit-testing programmes may not be feasible.

## Conclusions

Targeted universal use of N95 respirators is associated with effective control of hospital SARS-CoV-2 outbreaks regardless of initial size or variability across wards and infection sources, balancing protection with efficiency and comfort. We recommend flexible mask policies that switch between N95 respirators and surgical masks based on risk levels, optimising safety and resources in protecting healthcare workers and patients.

## Author contributions

**Mari Yanaka:** Writing – review & editing, Methodology, Data curation, Conceptualization. **Toshibumi Taniguchi:** Writing – original draft, Methodology, Investigation, Formal analysis. **Misuzu Yahaba:** Writing – review & editing, Data curation. **Shota Murata:** Writing – review & editing, Resources, Data curation. **Hiroshi Yoshikawa:** Writing – review & editing, Data curation. **Hitoshi Chiba:** Writing – review & editing, Supervision. **Misao Urushihara:** Resources, Data curation. **Hidetoshi Igari:** Writing – review & editing, Supervision.

## Ethics approval

This study was approved by the Chiba University Hospital Institutional Review Board (approval No. HK202404-11).

## Funding sources

This research did not receive any specific grant from funding agencies in the public, commercial, or not-for-profit sectors.

## Conflict of interest statement

The authors declare no potential conflicts of interest with respect to the research, authorship, and/or publication of this article.
