# Supplementary material for: Minimising the duration of N95 respirator use during hospital SARS-CoV-2 outbreaks: A mixed-effects analysis of post-screening infection reduction
Source: Infect Prev Pract. 2025 Sep 8;7(4):100484. doi: 10.1016/j.infpip.2025.100484 (PMC12482620; doi:10.1016/j.infpip.2025.100484)
Supplement: Multimedia component 1 [file mmc1.docx]

**Supplementary Material**

**Minimising the Duration of N95 Respirator Use During Hospital SARS-CoV-2 Outbreaks: A Mixed-Effects Analysis of Post-Screening Infection Reduction**

Mari Yanaka^1^, Toshibumi Taniguchi^1^, Misuzu Yahaba^1^, Shota Murata^2^, Hiroshi Yoshikawa^1^, Hitoshi Chiba^1^, Misao Urushihara^1^, Hidetoshi Igari^1^

^1^Department of Infectious Disease, Chiba University Hospital

^2^Division of Laboratory Medicine, Chiba University Hospital

| **Parameter** | **Value** |
| --- | --- |
| **Point Estimates** | |
| Mean difference (N95 - Surgical) | -3.020 |
| Median difference | -3.025 |
| Standard deviation | 1.498 |
| **Effect Size** | |
| Cohen's d | -0.931 |
| **Confidence Intervals** | |
| 95% CI (Percentile method) | [-5.975, -0.083] |
| 95% CI (BCa method) | [-5.975, -0.083] |
| **Probability Estimates** | |
| P (N95 reduces infections) | 97.8% |
| P (reduction ≥ 2 cases) | 74.7% |
| **Clinical Impact** | |
| Number Needed to Treat (clusters) | 4.3 |
| **Distribution Properties** | |
| Shapiro-Wilk W | 0.9996 |
| Shapiro-Wilk p-value | 0.4675 |
| Distribution | Approximately normal |

**Table S1. Bootstrap Analysis Results (n = 10,000 iterations)**

*BCa: Bias-Corrected and accelerated





**Figure S1. SARS-CoV-2 Cluster Outbreaks in Hospital, 2021–2024.** This graph shows 18 SARS-CoV-2 cluster outbreaks in Chiba University Hospital over the study period. Each point represents an outbreak, with size indicating cluster size (total number of infections). Red points indicate outbreaks managed with surgical masks only, and blue points indicate outbreaks managed with universal N95 respirators. The y-axis shows new infections detected after screening per outbreak. The temporal distribution shows the implementation of the N95 protocol beginning in August 2022.
